# Myoclonus in geriatric dogs and its association with canine cognitive dysfunction: an online survey

**DOI:** 10.3389/fvets.2026.1745264

**Published:** 2026-02-26

**Authors:** Samira Moana Brühl, Holger Andreas Volk, Karl Rohn, Nina Meyerhoff

**Affiliations:** 1 Department of Small Animal Medicine and Surgery, University of Veterinary Medicine Hannover, Foundation, Hannover, Germany; 2 Frontier Referral Center, Hergolding, Germany; 3 Department for Biometry, Epidemiology and Information Processing, University of Veterinary Medicine Hannover, Foundation, Hannover, Germany

**Keywords:** canine cognitive dysfunction, descriptive statistic, myoclonus, online survey, owner questionnaire

## Abstract

**Background:**

An increasing number of dogs are presented with suspected canine cognitive dysfunction (CCD), and a subset also exhibits myoclonus.

**Objectives:**

Because CCD shares multiple pathological and pathophysiological features with Alzheimer's disease in humans, and myoclonus has been linked to neurodegenerative disorders in people, the aim of the study was to describe myoclonus in dogs with clinical signs of CCD.

**Material and methods:**

An anonymous online survey for owners of geriatric dogs (over 7 years of age), consisting of 46 questions, was conducted. The survey included items on signalment, the Canine Dementia Scale (CADES), and myoclonus. CCD was defined based on a CADES score of >8. It was available online in both German and English from April to June 2024.

**Results:**

Of the 401 participants, 148 dogs were excluded due their young age, under 7 years and incomplete CADES-Score. Among the remaining respondents, 89% of owners reported that their dog showed signs of CCD. Overall, 146/164 (89.0%) dogs exhibited both CADES-defined CCD and myoclonus. However, no statistical significance was found between the co-occurrence of CCD screen status and the presence of myoclonus (*p* = 0.45). Predominantly, myoclonus occurred spontaneously (72.6%, *n* = 119), stress-induced (15.2%, *n* = 25), and light-induced (11.6%, *n* = 19). Noise-induced and feeding-induced myoclonus were the least common trigger (3.0%, *n* = 5 each), as well as no answer given (0.6%, *n* = 1). Ten dogs had multiple trigger causes.

**Clinical significance:**

Myoclonus was commonly co-reported alongside CADES-defined CCD by respondents. Although no statistically significant association between CCD and myoclonus was detected, CCD should be considered among the differential diagnosis in geriatric dogs presenting with myoclonus, particularly in the context of concurrent cognitive decline.

## 1 Introduction

Canine Cognitive Dysfunction (CCD), which shares several pathophysiological similarities with Alzheimer's disease (AD) in humans, is a prevalent age-related neurodegenerative condition in dogs (1–3). The prevalence of CCD increases with advancing age, beginning at approximately 8 years of age with reported prevalence rates ranging from 14%−35.0% in dogs over 8 years old (1, 4, 5). In dogs aged 11–12 years, prevalence reaches approximately 28.0% and exceeds 68.0% in dogs aged 15–16 years (4–7). Given the growing geriatric dog population, CCD represents an increasingly relevant clinical and welfare concern.

Unlike humans, no genetic risk factors, such as mutations in amyloid precursor protein (APP) and presenilin 1/2 have been identified in dogs as contributing to CCD (8, 9). Common risk factors for CCD in dogs include poor oral and dental health, lower body weight in kilogram (kg), increasing age and idiopathic epilepsy (1, 7, 10–12). Age-related immune decline contributes to cumulative cellular damage and chronic inflammation (11). The pathophysiology of CCD is multifactorial, with one key alteration being the deposition of toxic amyloid-beta proteins in the brain (1, 4, 13, 14), reduced concentrations of neurotransmitters such as dopamine, acetylcholine, norepinephrine, and gamma-aminobutyric acid (GABA) (1, 15, 16), mitochondrial dysfunction (1, 4, 17) as well as further mechanisms like neuroinflammation and vascular dysfunction (1, 14). Due to the shared pathological mechanisms, CCD and AD exhibit numerous parallels, positioning the dog as a potential model for AD (1, 3, 4, 14, 18). However, a notable difference is the absence of neurofibrillary tangles (NFTs) in dogs, a hallmark of AD in humans, possibly reflecting species-specific differences or the shorter canine lifespan (1, 19). Clinical signs of dogs with CCD have been summarized using the DISHAA acronym in dogs (Disorientation, Social Interaction changes, Sleep-Wake cycle disturbances, Learning and Memory alterations, Activity level decline, and Anxiety increase) (4, 20).

Myoclonus describes involuntary movements characterized by sudden, brief muscle contractions affecting one or multiple muscle groups (19, 21, 22). These typically arrhythmic, shock-like jerks last 20–250 milliseconds and may arise from sudden muscle contractions or relaxation (21, 23, 24). Myoclonus can originate from cortical, subcortical, and spinal sources, an overview of myoclonus classifications in dogs is provided in Table 1. In dogs, myoclonus has been described in a variety of clinical contexts, including epilepsy (25), metabolic or toxic encephalopathies (26), infectious or inflammatory diseases (21, 26, 27), idiopathic (28), and degenerative neurological conditions (29, 30), indicating that myoclonus represents a non-specific neurological sign rather than a disease entity. In human medicine, neurodegenerative disorders such as Alzheimer's disease are associated with cortical network hyperexcitability, impaired excitation–inhibition neurotransmitter balance, and synaptic dysfunction, which have been linked to the occurrence of myoclonus and epileptic seizures (19, 31–35). Comparable neuropathological features, including amyloid-β accumulation, synaptic pathology, and age-related neuronal network alterations, have been documented in dogs with canine cognitive dysfunction (4). Although a direct causal relationship cannot be established based on the present data, these shared neurodegenerative mechanisms may plausibly contribute to network instability and facilitate abnormal involuntary movements such as myoclonus in affected dogs.

**Table 1:** Classification criteria of myoclonus in dogs with its different subcategories.

**Classification- criteria**	**Subcatergories**
Nature (21, 25, 26)	Physiological
	Pathological
Type (25)	Positive = muscle contraction due to excitatory input
	Negative = sudden muscle tone loss due to inhibitory input
Origin (21, 25, 26)	Cortical
	Subcortical
	Spinal
	Brainstem
Epileptic nature (21, 25, 26)	Epileptic
	Non-epileptic
Timing (21, 25, 26)	Action-related
	At rest
Distribution (21, 25, 26)	Segmental
	Focal
	Multifocal

To date, a potential association between myoclonus and geriatric age has primarily been described in Cavalier King Charles Spaniels (CKCS), where myoclonus has been reported alongside behavioral changes suggestive of CCD (30, 36). Together, these observations suggest a plausible conceptual link between CCD-related neurodegeneration and the occurrence of myoclonus in geriatric dogs, although this relationship has not yet been systematically investigated.

Data regarding the co-occurrence of CCD and myoclonus in geriatric dogs remain limited. Therefore, this cross-sectional, survey-based study aims to promote recognition and awareness of the combined occurrence of CCD and myoclonus in an aging dogs, to provide an overview of CCD distribution, and to explore features associated with CCD onset to refine clinical assessment.

## 2 Material and methods

An anonymous online survey was conducted from April 2024 to June 2024 using LimeSurvey, available in both English and German. The survey was distributed via social media channels, including Facebook and Instagram, as well as on the animal hospital's homepage. The study's focus on CCD and myoclonus was communicated to the dog owners in advance (Supplementary File 1). Owners provided informed consent and agreed to privacy policies before entering information into the online questionnaire.

The survey (Supplementary File 1) included 46 questions across six categories: signalment, spatial orientation, social interaction, sleep-wake cycle, house-soiling, and myoclonus. It collected information on the owner (country, state, language) and the dog's signalment and medical history. For the purposes of this study, canines of all breeds and ages were considered geriatric if they had reached the age of seven, and thus were included in the study. Dogs were eligible for inclusion if they were aged 7 years or older and had a completed CADES score. The survey incorporated questions from the validated Canine Dementia Scale (CADES), which categorizes dogs into four groups: normal aging, mild, moderate, and severe CCD (20). Based on the CADES scores, participants were subsequently divided into two major groups: CADES-positive (score > 8) and CADES-negative (score 0–7). Additional specific questions on myoclonus, including its timing, frequency, affected body parts, occurrence of additional general tonic-clonic seizures (GTCS), medication use, improvement rate, and any further diagnostic investigations were asked. To facilitate recognition of myoclonus, a video of a Cairn Terrier (Supplementary Video 1) demonstrating myoclonus was included. In addition, the term “myoclonus” was defined as “a sequence of repeated, often arrhythmic, brief, shock-like jerks due to the sudden contraction or relaxation of one or more muscles.” This definition was provided to dog owners to facilitate recognition of myoclonus-like movements and to support differentiation from other abnormal movements. For data analysis, the questionnaire terms “myoclonus,” “myoclonic twitches,” “myoclonic seizures,” “shrinking back,” and “twitches” were consolidated under the single category “myoclonus.” Generalized tonic–clonic seizures were evaluated separately and were described to dog owners using typical clinical signs, including loss of consciousness, limb paddling or rigidity, involuntary urination or defecation, postictal disorientation, and transient blindness.

The owner of the Cairn Terrier signed a data privacy statement and a consent form for uploading this video to the survey as well as to social media. Data was collected via LimeSurvey^®^ and compiled in Microsoft Excel (Microsoft 365, Version 2401). The statistical analyses were conducted using the statistical software SAS^®^, Version 9.4M7 [OS: WIN (X64_SRV19)] through SAS Studio 3.81 (SAS Institute, Cary, NC, USA) (Release year 2020), and JASP Team (2025), JASP (Version 0.95.3).

Statistical analysis comprised descriptive statistics with simple frequencies, distribution analyses, Spearman correlation analyses (CADES-Score, year of birth, weight (in kg) and age of onset of myoclonus), and two-tailed chi-squared tests (sex vs. CCD). The primary analysis evaluated the co-occurrence between CCD (CCD-positive vs. CCD-negative) and the presence of myoclonus using a two-tailed chi-square test and Fisher's exact test for small sample sizes. In order to assess the association between the occurrence of myoclonus and the CADES score, a logistic regression analysis was performed with the binary outcome variable myoclonus (yes/no) and the CADES score as the independent variable. Additionally, a Mann-Whitney-U-test has been used to compare CADES scores between dogs with and without myoclonus to explore associations with disease severity.

The null hypothesis postulated no association between CADES status (positive vs. negative) and the co-occurrence of myoclonus, whereas the alternative hypothesis postulated an association between CADES status and the presence of myoclonus.

A *p-value* ≤ 0.05 was considered statistically significant and led to rejection of the null hypothesis.

## 3 Results

### 3.1 General information

**Figure 1:**
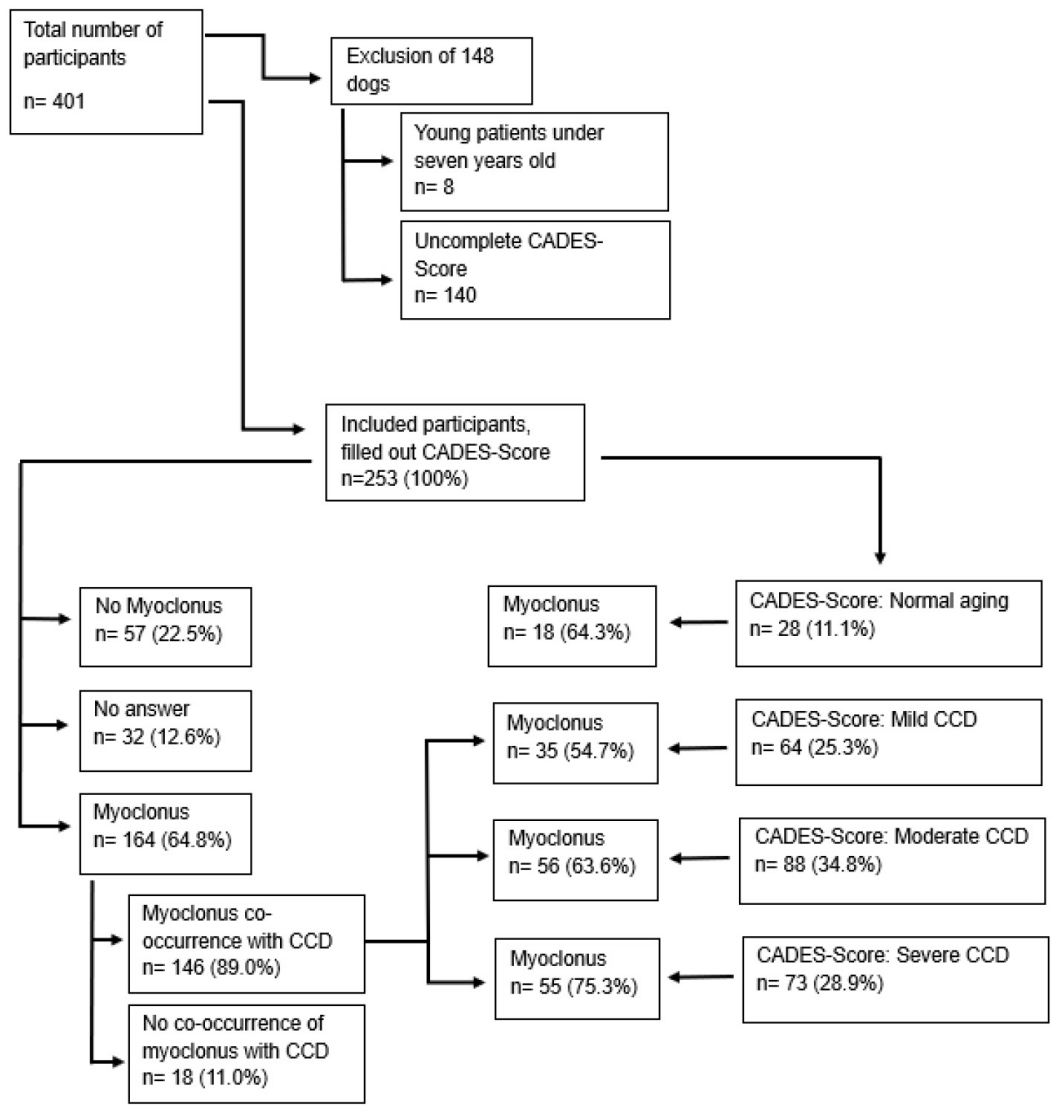
Summary of included and excluded dogs, number of dogs with canine cognitive dysfunction (CCD) and absolute number of dogs with myoclonus. CADES, Canine Dementia Scale.

A total of 401 dog owners participated in the survey. Of these, 148 responses were excluded because the dogs were younger than seven years or the CADES-Score was incomplete (Figure 1). The following results were based on the remaining 253 responses. A total of 220 (87.0%) respondents completed the survey in German, while 33 (13.0%) in English. The majority of participants were from Germany (82.0%), followed by the USA (8.7%) and Austria (3.6%). The remaining 5.7% were distributed across 10 other countries or did not specify their country.

### 3.2 Signalment of the study population

A total of 47 dog breeds were represented in the survey. The most common breed were mixed breed (38.3%, *n* = 97), followed by Retriever (Labrador/Golden) which were summarized in the questionnaire (11.9%, *n* = 30), Terrier (8.7%, *n* = 22), Border Collie (4.0%, *n* = 10), Australian Shepherd and Dachshund (3.2%, each *n* = 8), Beagle, Cocker Spaniel, CKCS, and Poodle (2.4% each, *n* = 6), Giant Schnauzer (1.2%, *n* = 3), and Bavarian Mountain Hound (0.4%, *n* = 1). Several other breeds were included (19.8%, *n* = 50), each represented by only a few individuals, and were not analyzed separately. The study cohort primarily consisted of neutered males (43.5%, *n* = 110) and spayed females (42.3%, *n* = 107), as well as intact females (4.7%, *n* = 12) and intact males (9.1%, *n* = 23). One dog (0.4%) had no sex recorded. The average weight was 18.0 kg (range, 2.7–55.0 kg). The year of birth was reported to allow for the accurate calculation of participants' age at the time of survey completion. The median age of dogs at time of study was 14 years (range 7–24 years).

### 3.3 Medical history

None-cognitive health issues were reported in 180 (71.1%) dogs. Among the 225 dogs in the CADES-positive group, 161 (71.6%) showed non-cognitive comorbidities, of which 64/225 (28.4%) had orthopedic complaints, 48/225 (21.3%) heart diseases and 36/225 (16.0%) endocrine disorders. Epilepsy was diagnosed in 11/225 (4.9%) dogs. One hundred and ten of the general CADES positive dogs (48.9%) were suffering from more than one health issue.

In the CADES negative group (*n* = 28), 19/28 dogs (67.9%) had non-cognitive health issues, with the most common being orthopedic complaints 10/28 (35.7%), followed by endocrine disorders 3/28 (10.7%), and heart diseases 2/28 (7.1%), 2/28). Epilepsy was present in 2 dogs (7.1%). Six dogs out of 28 (21.4%) had more than one health issue.

Long-term medication or supplements was reported for 183/253 (72.3%) dogs, whereas 29 (11.5%) dogs did not require long-term medications, and 41 cases (16.2%) had unknown medication status. Among the 183 dogs receiving long-term treatment, medications included propentofylline (30.1%, *n* = 55), pimobendane (15.8%, *n* = 29), levothyroxine (14.2%, *n* = 26), bedinvetmab (12.0%, *n* = 22), gabapentinoids (10.4%, *n* = 19), non-steroidal anti-inflammatory drugs (NSAIDs) (8.2%, *n* = 15), B vitamins (7.7%, *n* = 14), corticosteroids (4.9%, *n* = 9), and cannabidiol (CBD) (3.8%, *n* = 7).

### 3.4 Cognitive dysfunction

**Figure 2:**
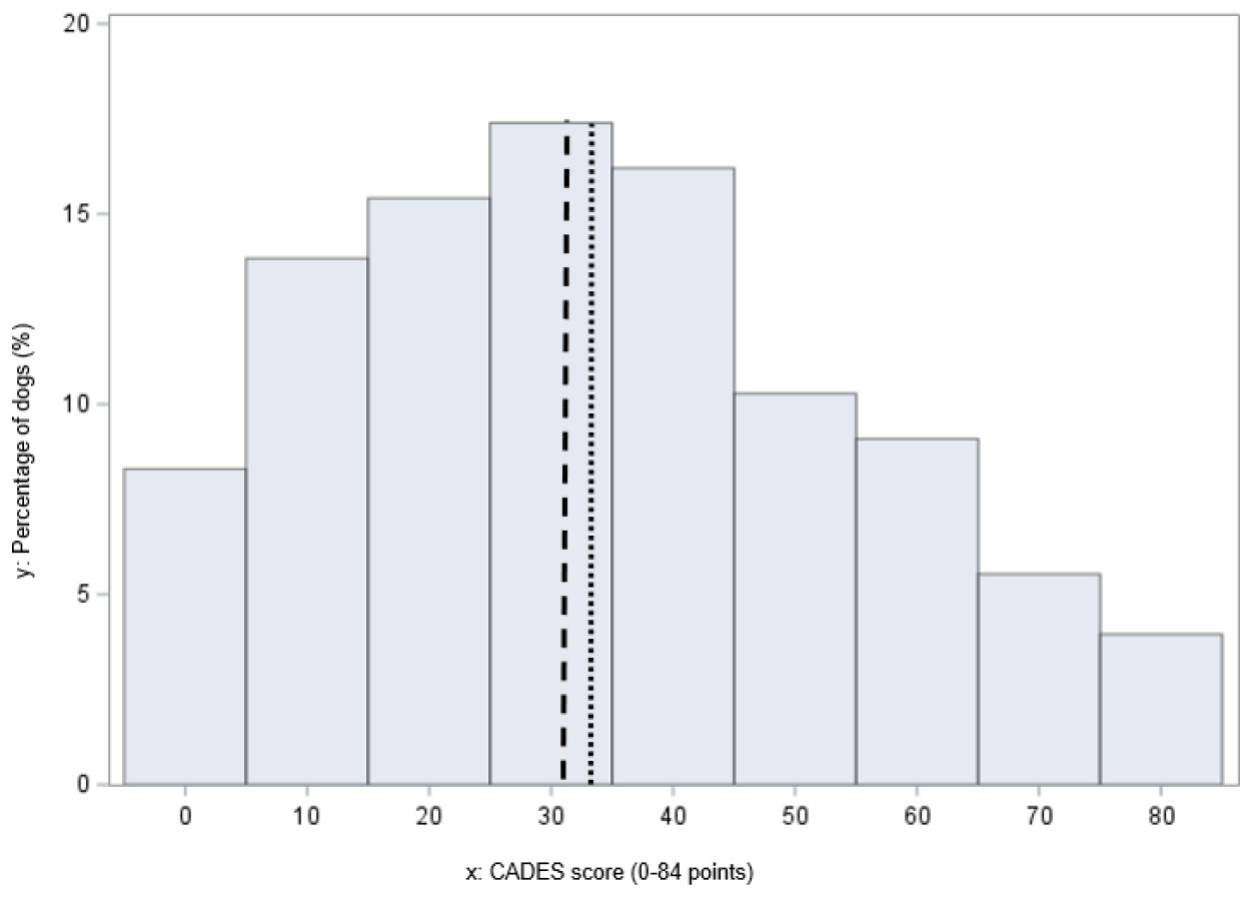
Histogram shows CADES (Canine Dementia Scale) scores in 253 dogs. Mean = 33 (….), median = 31 (– –). Higher scores indicate greater cognitive impairment, mean score 33, median score 31 (range 0–84 points).

In 80.2% (*n* = 203) of cases, dog owners suspected that their dog was exhibiting clinical signs of CCD prior to completing the CADES-questionnaire, whereas 17.4% (*n* = 44) did not report this suspicion, and 2.4% (*n* = 6) did not provide a response. The completed CADES scores, based on 253 responses, ranged from 0 to 84 points, with a mean score of 33 and a median score of 31 (Figure 2). Regarding CADES, 28.9% (*n* = 73) of dogs were classified as severe CCD, 34.8% (*n* = 88) dogs as moderate CCD, 25.3% (*n* = 64) as mild CCD and 11.1% (*n* = 28) showed no signs of CCD (Figure 1). Among CADES-defined CCD cases, 28.9% (*n* = 65) had exhibited signs for 2–6 months, 26.7% (*n* = 60) for 12 months and 25.8% (*n* = 58) for 7–12 months. In 5.8% (*n* = 13), dog owners reported onset 1–4 weeks before completing the questionnaire. The most frequently reported clinical signs were disorientation (31.1%, *n* = 70), myoclonus (23.1%, *n* = 52), and anxiety (12.9%, *n* = 29), as indicated in response to the “first signs” item (A13 questionnaire, Supplementary File 1). Dogs with epilepsy had a median CADES score of 61 (range 9–84), whereas dogs without epilepsy had a median CADES score of 35 (range 8–81). A Mann–Whitney U-test was performed to compare CADES-positive dogs with and without epilepsy. Although dogs with epilepsy tended to have higher CADES scores, this difference did not reach statistical significance (*U* = 1387, *p* = 0.32). A chi-square test and Fisher's exact test was employed to compare the occurrence of epilepsy in CCD positive and negative groups. This value yielded *x*^2^ = 0.0031 (df = 1, *p* < 0.956) (Fisher's exact test: OR = 0.67, *p* = 0.64), indicating no statistical significance. According to the Spearman correlation, a weak negative correlation (−0.328) was revealed between CADES scores and body weight (in kg), indicating that individuals with lower body weight (in kg) tended to have higher CADES scores (*p* < 0.0001). Additionally, a weak negative correlation (−0.288) was observed with the year of birth, indicating that earlier birth years are associated with higher CADES scores (*p* < 0.0001). The age at onset of myoclonus was weakly positively correlated (0.317) with a higher CADES score (*p* < 0.0001; Table 2).

**Table 2:** Spearman-correlation between CADES (Canine Dementia Scale) and weight in kg, year of birth, and age in years at first myoclonus.

**Spearman correlation p-value number of observations**	
	**CADES_score**
*Weight in kg*	−0.32869 < 0.0001
Year of birth	−0.28840 < 0.0001
Age of onset of myoclonus	0.31764 < 0.0001

The correlation between weight, year of birth and CADES is weakly negatively correlated. The age of onset of myoclonus and CADES is a weakly positive correlate.

To assess differences between sex (female vs. male) and the occurrence of CCD a chi-square test and Fisher's exact test was performed. This value yielded *x*^2^ = 0.098 (df = 1, *p* < 0.755). Fisher's exact test revealed no statistical significance (*p* < 0.842) between the groups. The log odds ratio was −0.125 (95% CI: −0.992 to 0.742) indicating no significant effect.

### 3.5 Myoclonus

Overall, myoclonus was reported in 64.8% (*n* = 164) of dogs, whereas 22.5% (*n* = 57) had no myoclonus, and 12.6% (*n* = 32) of responses were missing. Among dogs with myoclonus, 146/164 (89.0%) were CADES-positive, while 18/164 (11.0%) exhibited myoclonus without CCD signs (Figure 1). The reported median age at initial onset of myoclonus was 12 years (range: 1–18 years). Myoclonus occurred spontaneously in 72.6% of cases (*n* = 119), followed by stress-induced (15.2%, *n* = 25) and light-induced (11.6%, *n* = 19). Noise-induced and feeding-induced myoclonus were the least common (3.0%, *n* = 5 each), as well as no answer given (0.6%, *n* = 1). Ten dogs (6.1%) had multiple triggers. Myoclonus primarily occurred during the day (73.2%, *n* = 120), followed by the evening (16.5%, *n* = 27) and morning (4.3%, *n* = 7). In nine (5.5%) cases, the time of onset remains unknown. Episodes were most commonly observed at rest (56.7%, *n* = 93), followed by walks (15.9%, *n* = 26) and stressful situations (15.2%, *n* = 25). In 20 cases (12.2%), the situation remains unclear due to missing responses. Myoclonus affected multiple body parts (head, pelvic limbs, thoracic limbs, neck, trunk) in 80 cases (48.8%), and a single body part in 75 cases (45.7%), with the head being (23.2%, *n* = 38) most commonly affected. In nine dogs (5.5%), the region of myoclonus remains unknown. Myoclonus episodes occurred multiple times daily (42.7%, *n* = 70), multiple times weekly (32.3%, *n* = 53), once monthly (10.4%, *n* = 17), and daily occurrences being the least frequent (2.4%, *n* = 4). Frequency data were not missing in 20 cases (12.2%).

Twenty-three dogs received medication for myoclonus, including propentofylline (7/23), gabapentin (6/23), phenobarbital (2/23), corticosteroids (2/23), levetiracetam (1/23), imepitoin (1/23), CBD (1/23), B vitamins (1/23), and snake venom enzymes (1/23), unknown medication (3/23). Two dogs were on two medications simultaneously. Owners reported improvement in 17/23 cases, in 52.2% (*n* = 12) severity and frequency were reduced, in 17.4% (*n* = 4), myoclonus resolved completely, in 4.3% (*n* = 1) data is missing. Resolution occurred with corticosteroids or gabapentin. Improvement was observed within a week in 26.1% (*n* = 6) of cases, immediately after the first dose in 17.4% (*n* = 4), after two dose administration in 4.3% (*n* = 1), and within 1 month 4.3% (*n* = 1). Data were not reported in 26.1% (*n* = 6). The most common additional diagnostic for myoclonic work-up were routine blood work (9.1%, *n* = 15), followed by electroencephalogram (EEG) (1.2%, *n* = 2), magnetic resonance imaging (MRI) (0.6%, *n* = 1), and genetic testing (0.6%, *n* = 1). Most dogs underwent no further diagnostics (72.6%, *n* = 119), and in 15.9% (*n* = 26) the diagnostic work-up was unknown.

**Figure 3:**
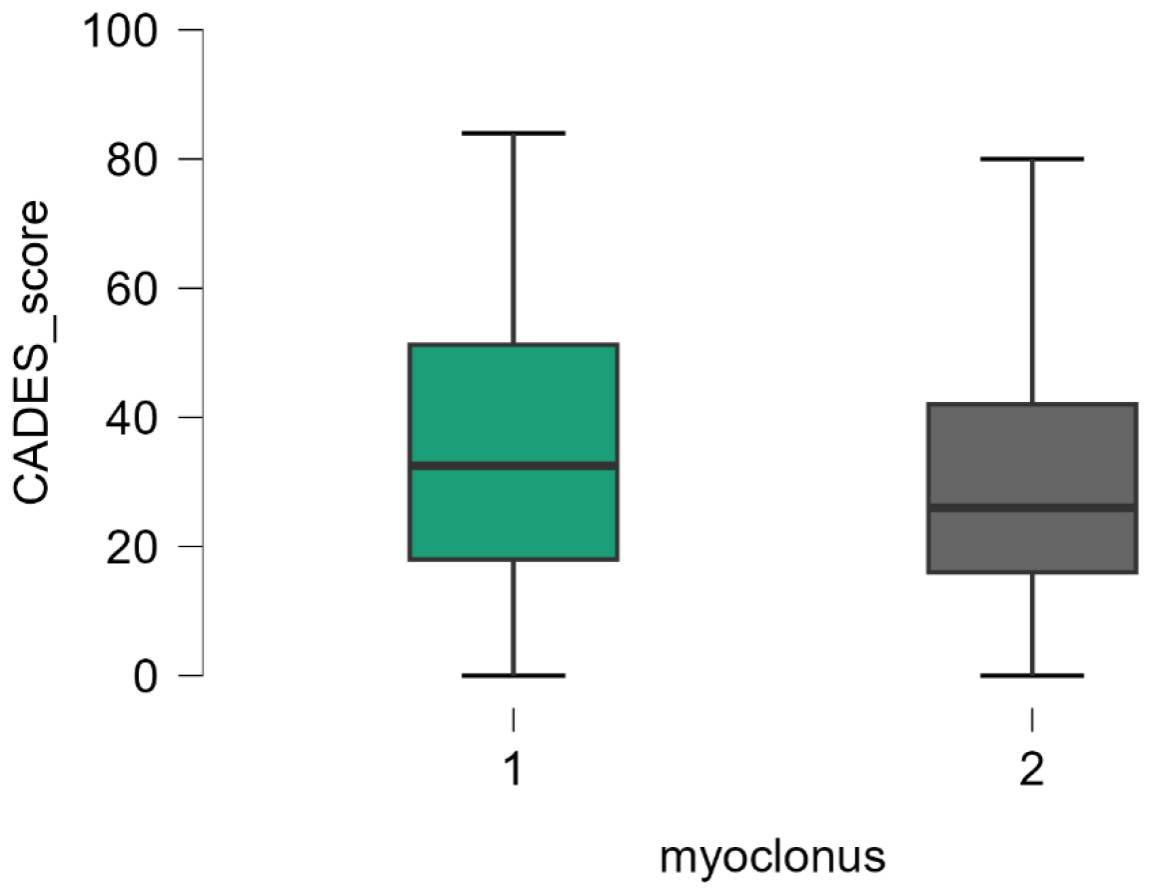
Box-plot CADES score and myoclonus. No statistically significant difference was found between the two groups (*p* = 0.055) using Mann-Whitney U-test to verify the dependence of the CADES severity score and myoclonus, x-axis: 1 = myoclonus present; 2 = myoclonus not present; y-axis: CADES-Score (Canine dementia Scale).

A total of 146 dogs exhibited the simultaneous occurrence of signs of CCD, a positive CADES score and myoclonus. Meanwhile, 18 dogs had no positive CADES score but still experienced myoclonus. To enable better comparison of these groups, a chi-square test and Fisher's exact test was performed on these variables. This value yielded *x*^2^ = 0.739 (df = 1, *p* < 0.39; Table 3). No statistical significant association was observed between CCD screen status and the presence of myoclonus in the study population (Fisher's exact test, *p* = 0.45). The odds of myoclonus in CCD-positive dogs were 0.61 times those in CCD-negative dogs (OR = 0.61, 95% CI: 0.14–1.98). Dogs exhibiting myoclonus had a mean CADES score of 35 points (range: 0–84 points), whereas dogs without myoclonus showed a mean CADES score of 30 points (range: 0–80 points) (Figure 3, *p* = 0.055). In dogs where myoclonus occurred, 5% of the CADES score values were less than 3, while 95% of the values were less than 72. In dogs where myoclonus did not occur, 5% of the values were equal to 0, and 95% of the CADES score values were less than 77.

**Table 3:** Contingency table for the application of the Chi-square test and Fisher's exact test to compare the occurrence of myoclonus (yes/no) between CCD (Canine cognitive dysfunction)-positive and negative dogs.

	**CCD positive**	**CCD negative**	
Myoclonus yes	146	18	164
Myoclonus no	53	4	57
	199	22	221
**Chi-squared tests**
Value	df	*p*
*X^2^*	0.739	1	3.899 × 10^−1^
*N*	221		
	*Log odds ratio*
	Log odds ratio	95% confidence intervals		
		Lower	Upper	*p*
Odds ratio	−0.491	−1.619	0.637	
Fisher's exact test	−0.489	−1.935	0.683	4.542 × 10^−1^

P-value shows no statistical significance (p = 0.45).

To compare the occurrence of myoclonus (independent variable) on the CADES severity score (dependent variable), the Mann-Whitney U-test was conducted for these two variables. The U-statistic yielded a value of 5,337. Since the *p-value* (*p* = 0.055) is greater than the significance level (alpha ≤ 0.05), the results are not statistically significant, but a trend could be assumed.

**Figure 4:**
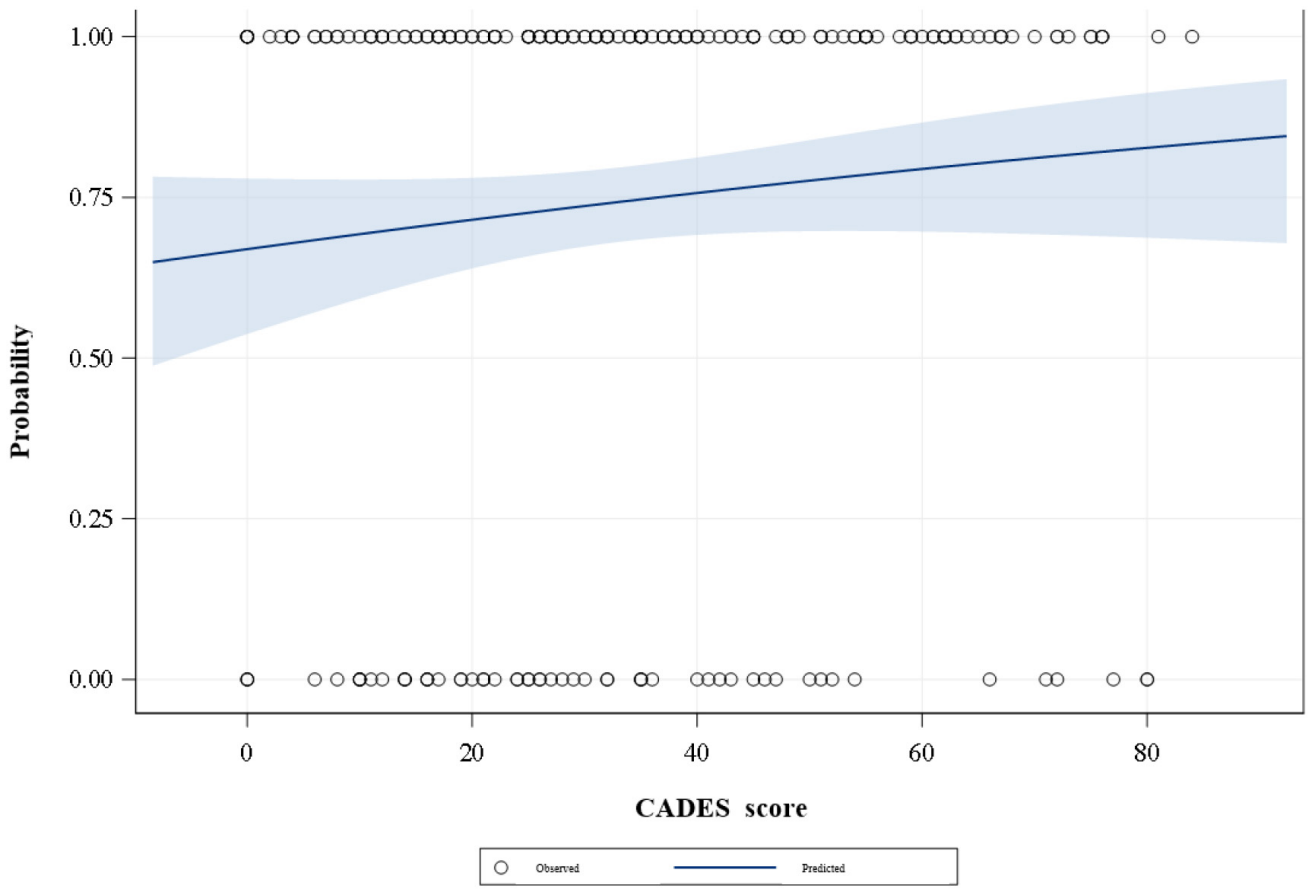
Logistic regression analysis of the association between the CADES score and myoclonus. The CADES score is shown on the x-axis, and the predicted probability of myoclonus on the y-axis. White circles represent observed myoclonus outcomes, and the blue line depicts the fitted probabilities derived from the logistic regression model.

The logistic regression analysis, with myoclonus as the dependent variable and the CADES score as the independent variable, showed no statistically significant association between the CADES score and the occurrence of myoclonus [odds ratio (OR) = 1.01, 95% confidence interval (CI) 0.996–1.026, *p* = 0.15] (Figure 4). Accordingly, there was no evidence that the CADES score affected the likelihood of developing myoclonus.

## 4 Discussion

The study describes a population of 253 dogs in which owners suspected CCD, myoclonus or both. To the author's knowledge, the co-reporting of myoclonus in dogs with suspected CCD has not been previously described. This survey demonstrates that myoclonus may co-occur with CADES-defined CCD. Myoclonus was frequently co-reported in dogs with CADES-defined CCD, with 89.0% of dogs showing both CCD-related signs and myoclonus. However, owners were informed that the survey focused on CCD and myoclonus, which may have influenced reporting. This participant-facing framing could introduce selection and priming biases, potentially inflating co-reporting rates. Therefore, the observed co-occurrence should be interpreted with caution and may not reflect the true prevalence in the general population. Although myoclonus was reported more frequently in dogs with CADES-defined CCD, there was no statistical significant association between CCD and myoclonus, using logistic regression (OR = 1.01, 95% CI: 0.996–1.02, *p* = 0.15). In human medicine, it has long been known that myoclonus can occur concurrently with neurodegenerative disorders, particularly in AD (32, 37). Comparable neuropathological changes, including amyloid-β accumulation and synaptic pathology, have been described in dogs with CCD (1, 4). In this study the most frequently reported clinical signs in CCD-affected dogs were disorientation (31.1%) and myoclonus (23%). While these findings indicate that myoclonus may be a commonly co-reported clinical feature of CCD, the cross-sectional and survey-based design of this study prevents any conclusions regarding causality or predictive value. Dog owners and veterinarians should be aware that myoclonus may be a co-reported clinical sign of CADES-defined CCD.

For the evaluation of CCD, CADES score was selected as a validated questionnaire due to its high sensitivity, particularly in capturing the transitions between normal aging to mild and severe cognitive changes (38, 39). In the present study, 225 dogs scored CADES-positive, with scores ranging from mild to severe, with an average score of 33.

Myoclonus is defined as an involuntary movement which is characterized by sudden, brief muscle contractions. As detailed in Table 1, an important factor is the differentiation between epileptic and non-epileptic myoclonus. Whereas, myoclonic epilepsy tends to show a synchronized cortical discharge of action potentials with synaptic interactions, non-epileptic myoclonus arises from hyperexcitability due to neuronal lesions or degenerative processes (40, 41). Clinically, absence of GTCS, may help distinguish non-epileptic myoclonus in both veterinary (22) and human medicine (42, 43), although cortical myoclonic epilepsy (22) cannot be entirely ruled out. For accurate differentiation, an EEG examination is necessary to determine whether the origin is cortical or subcortical (22). In the present study, only 11/225 CCD-positive dogs had a diagnosis of epilepsy, thus a non-epileptic origin of myoclonus is considered most plausible. However, the lack of EEG and other diagnostics prevents definitive classification.

Future studies should include a comprehensive diagnostic work-up of CCD dogs with myoclonus, including completion of the CADES score, exclusion of metabolic [e.g., hypoglycemia (44)], structural, iatrogenic (45) and genetic causes in predisposed breeds [e.g., Lafora disease (46)] through laboratory testing and brain MRI. Furthermore, EEG examination is recommended to enable a more precise differentiation of the origin of myoclonus.

Previously recognized risk factors for CCD include idiopathic epilepsy (12), body weight in kg and increasing age (1, 7). Considering the current sample size, no statistically significant difference was observed in either the co-occurrence of CADES-positive dogs and pre-existing epilepsy or the CADES scores between dogs with and without previously reported epilepsy. Descriptively, however, dogs with pre-existing epilepsy tended to have higher CADES scores compared to dogs without epilepsy (61 vs. 35), suggesting that patients with pre-existing neurological impairment from epileptic seizures may be more prone to developing higher CADES scores, but further prospective studies are necessary to determine the clinical relevance of this observation (12, 47). In this study a significantly weak negative correlation between body weight (in kg) and CADES Score was found, as well as a statistical significant negative correlation between year of birth and CADES-Score, supporting previously reported data (1, 7).

Known causes of myoclonus in older dogs include Lafora disease in predisposed breeds (Dachshund, CKCS, Beagle) (29, 46, 48, 49), age-related CKCS-specific myoclonus (30, 36), and idiopathic myoclonic seizures in subsets of elderly dogs (28). Co-occurrence with CCD has not been systematically described prior to this study. In the present cohort the aforementioned breeds were rare (*n* = 20 in total), making Lafora disease or CKCS-specific myoclonus unlikely to account for the observed cases. Consistent with Lindner et al. (28), where 4 of 5 dogs developed myoclonus after 7 years of age, CCD may contribute to myoclonus in geriatric dogs, but this remains speculative, as CADES score were not assessed in that study.

Regarding possible therapeutic options, 23 owners reported administration of various medications for myoclonus, most commonly propentofylline or gabapentin, and one with levetiracetam. Observed changes in frequency and intensity were variable and cannot be interpreted as evidence of efficacy. Side effects of medication should always be considered, but no progression of myoclonus was observed in treated dogs. Controlled studies are required to evaluate therapeutic effects in dogs with CCD.

This study has several limitations. Survey results were based solely on owner-reported information, lacking confirmed veterinary diagnoses or comprehensive diagnostic evaluations, which may have resulted in false-positive or false-negative findings. The study population was biased toward dogs showing CCD-related signs and myoclonus, and respondents may not be representative for the overall dog population. Presenting a video illustrating myoclonus, combined with the challenge for dog owners to accurately distinguish myoclonus from other abnormal movements, may have contributed to priming, selection bias, and potential heterogeneous misinterpretation. Recall bias is possible due to the cross-sectional, non-prospective design. Participant-facing materials explicitly referenced CCD and myoclonus, further increasing the risk of reporting bias. Finally, the cross-sectional design prevented longitudinal assessment of myoclonus and CCD progression or long-term outcomes.

## 5 Conclusion

Myoclonus is common in geriatric dogs and can co-occur in CADES-defined CCD, but the observed co-occurrence may be influenced by survey design and reporting biases. Although no statistically significant association between the occurrence of myoclonus and CADES-defined CCD was identified, myoclonus may be a clinically observed concomitant feature in older dogs with CCD. Diagnostic work-up in geriatric dogs with CCD and myoclonus is limited and guidelines should be established in the future. Prospective veterinary-supervised studies are needed to clarify causality, track progression, and optimize combined therapy for CCD and myoclonus.

## Data Availability

The original contributions presented in the study are included in the article/Supplementary material, further inquiries can be directed to the corresponding author.

## Supplementary material

Supplementary File 1 Owner questionnaire and recruitment text for owner questionnaire.

Supplementary Video 1 Video Cairn Terrier with positive myoclonus.
