# Supplementary material for: Myoclonus in geriatric dogs and its association with canine cognitive dysfunction: an online survey
Source: Front Vet Sci. 2026 Feb 26;13:1745264. doi: 10.3389/fvets.2026.1745264 (PMC12980881; doi:10.3389/fvets.2026.1745264)
Supplement: Supplementary File 1 — Owner questionnaire and recruitment text for owner questionnaire. [file Supplementary_file_1.docx]

Supplementary Material

# Owner questionnaire

**Section A: Signalment**

- A1. What country are you from?
- A2. If you are from Germany, which federal state?
  - Hesse
  - Lower-Saxony
  - Schleswig-Holstein
  - North Rhine-Westphalia
  - Hamburg Bremen
  - Rhineland-Palatinate
  - Saxony
  - Saxony-Anhalt
  - Thuringia
  - Berlin
  - Brandenburg
  - Bavaria
  - Baden-Württemberg
  - Mecklenburg-Western Pomerania
  - Saarland
- A3. What type of pet do you own?
  - Dog
- A4. What breed does your pet belong to?
  - Beagle
  - Dachshound
  - Terrier
  - Australien Sheperd
  - Golden/Labrador Retriever
  - Boxer
  - Poodle
  - Mixed breed
  - Others:
- A5. What is the gender of your pet?
  - Female-Spayed
  - Female-intact
  - Male-intact
  - Male-neutered
- A6. When was your pet born (DD/MM/YYYY)
- A7. The current weight of your pet (in kg)?
- A8. Does your pet have known pre-existing conditions? (Heart, Kidney, Thyroid, Orthopedic problems, etc.)
  - Yes
  - No
- A9. If yes, which ones?
- A10. What continuous medication is your pet currently receiving?
- A11. Do you suspect that your pet is suffering from dementia?
  - Yes
  - No
- A12. Since when do you feel that your pet is suffering from dementia?
  - For a few weeks
  - For a few months
  - For half a year
  - For over one year
  - Other:
- A13. Which clinical signs did you notice first?
  - Cognitive Disorientation
  - Augmented anxiety states
  - Disturbance in Sleep-Wake Cycle
  - Diminished Interaction with owners/other conspecifics
  - Incontinence
  - Twitches or shrinking back (e.g. aversion to light)
  - Other:

**Section B: CADES: Spatial Orientation Canine Dementia Score**

- B1. Does your pet show disorientation in a familiar environment (indoors and outdoors)?
  - 0 Points: Abnormal behavior of the pet was never observed
  - 2 Points: Abnormal behavior of the pet was observed at least once in the last 6 months
  - 3 Points: Abnormal behavior occurred at least 1x per month
  - 4 Points: Abnormal behavior was observed 2-4x per month
  - 5 Points: Abnormal behavior was observed several times per week
- B2. Does your pet fail to recognize familiar persons & animals (indoors and outdoors)? B3. Does your pet show abnormal reactions to familiar objects (e.g., trash bin, chair)?
  - 0 Points: Abnormal behavior of the pet was never observed
  - 2 Points: Abnormal behavior of the pet was observed at least once in the last 6 months
  - 3 Points: Abnormal behavior occurred at least 1x per month
  - 4 Points: Abnormal behavior was observed 2-4x per month
  - 5 Points: Abnormal behavior was observed several times per week
- B4. Does your pet exhibit aimless wandering (restlessness during the day)?
  - 0 Points: Abnormal behavior of the pet was never observed
  - 2 Points: Abnormal behavior of the pet was observed at least once in the last 6 months
  - 3 Points: Abnormal behavior occurred at least 1x per month
  - 4 Points: Abnormal behavior was observed 2-4x per month
  - 5 Points: Abnormal behavior was observed several times per week
- B5.Does your pet show reduced ability to perform previously learned tasks or commands?
  - 0 Points: Abnormal behavior of the pet was never observed
  - 2 Points: Abnormal behavior of the pet was observed at least once in the last 6 months
  - 3 Points: Abnormal behavior occurred at least 1x per month
  - 4 Points: Abnormal behavior was observed 2-4x per month
  - 5 Points: Abnormal behavior was observed several times per week

**Section C: CADES: Social Interaction Canine Dementia Score**

- C1. Does your pet show changes in interaction between human and dog or dog and other dogs (playing, petting, greeting)?
  - 0 Points: Abnormal behavior of the pet was never observed
  - 2 Points: Abnormal behavior of the pet was observed at least once in the last 6 months
  - 3 Points: Abnormal behavior occurred at least 1x per month
  - 4 Points: Abnormal behavior was observed 2-4x per month
  - 5 Points: Abnormal behavior was observed several times per week
- C2. Are there changes in the individual behavior of the dog (exploration behavior, play, performance)?
  - 0 Points: Abnormal behavior of the pet was never observed
  - 2 Points: Abnormal behavior of the pet was observed at least once in the last 6 months
  - 3 Points: Abnormal behavior occurred at least 1x per month
  - 4 Points: Abnormal behavior was observed 2-4x per month
  - 5 Points: Abnormal behavior was observed several times per week
- C3. Does your pet show changes in the reaction to commands and the ability to learn new tasks?
  - 0 Points: Abnormal behavior of the pet was never observed
  - 2 Points: Abnormal behavior of the pet was observed at least once in the last 6 months
  - 3 Points: Abnormal behavior occurred at least 1x per month
  - 4 Points: Abnormal behavior was observed 2-4x per month
  - 5 Points: Abnormal behavior was observed several times per week
- C4. How irritable is your pet?
  - 0 Points: Abnormal behavior of the pet was never observed
  - 2 Points: Abnormal behavior of the pet was observed at least once in the last 6 months
  - 3 Points: Abnormal behavior occurred at least 1x per month
  - 4 Points: Abnormal behavior was observed 2-4x per month
  - 5 Points: Abnormal behavior was observed several times per week
- C5. Does your pet show new aggressive behavior
  - 0 Points: Abnormal behavior of the pet was never observed
  - 2 Points: Abnormal behavior of the pet was observed at least once in the last 6 months
  - 3 Points: Abnormal behavior occurred at least 1x per month
  - 4 Points: Abnormal behavior was observed 2-4x per month
  - 5 Points: Abnormal behavior was observed several times per week

**Section D: CADES: Sleep-Wake Cycles Canine Dementia Score**

- D1. Does your pet exhibit abnormal behavior at night (wandering, vocalizing, restless)?
  - 0 Points: Abnormal behavior of the pet was never observed
  - 2 Points: Abnormal behavior of the pet was observed at least once in the last 6 months
  - 3 Points: Abnormal behavior occurred at least 1x per month
  - 4 Points: Abnormal behavior was observed 2-4x per month
  - 5 Points: Abnormal behavior was observed several times per week
- D2. Is there a shift from insomnia to excessive sleep?
  - 0 Points: Abnormal behavior of the pet was never observed
  - 2 Points: Abnormal behavior of the pet was observed at least once in the last 6 months
  - 3 Points: Abnormal behavior occurred at least 1x per month
  - 4 Points: Abnormal behavior was observed 2-4x per month
  - 5 Points: Abnormal behavior was observed several times per week

**Section E: CADES: House Training Canine Dementia Score**

- E1. Does your pet deposit feces or urine at random locations at home?
  - 0 Points: Abnormal behavior of the pet was never observed
  - 2 Points: Abnormal behavior of the pet was observed at least once in the last 6 months
  - 3 Points: Abnormal behavior occurred at least 1x per month
  - 4 Points: Abnormal behavior was observed 2-4x per month
  - 5 Points: Abnormal behavior was observed several times per week
- E2. Is the feces/urine deposited in its kennel or at its sleeping place?
  - 0 Points: Abnormal behavior of the pet was never observed
  - 2 Points: Abnormal behavior of the pet was observed at least once in the last 6 months
  - 3 Points: Abnormal behavior occurred at least 1x per month
  - 4 Points: Abnormal behavior was observed 2-4x per month
  - 5 Points: Abnormal behavior was observed several times per week
- E3. Have there been changes in signaling when needing to defecate/urinate?
  - 0 Points: Abnormal behavior of the pet was never observed
  - 2 Points: Abnormal behavior of the pet was observed at least once in the last 6 months
  - 3 Points: Abnormal behavior occurred at least 1x per month
  - 4 Points: Abnormal behavior was observed 2-4x per month
  - 5 Points: Abnormal behavior was observed several times per week
- E4. Does your pet deposit feces/urine in the house shortly after an outdoor walk?
  - 0 Points: Abnormal behavior of the pet was never observed
  - 2 Points: Abnormal behavior of the pet was observed at least once in the last 6 months
  - 3 Points: Abnormal behavior occurred at least 1x per month
  - 4 Points: Abnormal behavior was observed 2-4x per month
  - 5 Points: Abnormal behavior was observed several times per week
- E5. Does your pet deposit feces/urine in unusual places other than usual (grass, concrete)?
  - 0 Points: Abnormal behavior of the pet was never observed
  - 2 Points: Abnormal behavior of the pet was observed at least once in the last 6 months
  - 3 Points: Abnormal behavior occurred at least 1x per month
  - 4 Points: Abnormal behavior was observed 2-4x per month
  - 5 Points: Abnormal behavior was observed several times per week

**Section F: Myoclonus**

- F1. Does your pet show myoclonus? *Definition Myoclonus: sequence of repeated, often arrhythmic, brief shock-like jerks due to sudden involuntary contraction or relaxation of one or more muscle*
  - Yes
  - No
- F2. At what age did your animal first exhibit myoclonus?
- F3. How are the myoclonic twitches triggered in your pet? (as in the video)
  Noise-induced (For example, moving a metal bowl)
  - Light-induced (For example, sunlight, entering from the dark hallway into the bright living room)
  - Stress-induced
  - Spontaneous, without any specific reason
  - Other:
- F4. At what time of day do the myoclonic seizures occur most frequently?
  - In the early morning hours
  - Throughout the day
  - In the evening/At night
- F5. When do the twitches occur more frequently?
  - At rest
  - During walks
  - During stressful situations
  - Other:
- F6. Which body parts are most affected during the twitches (multiple answers possible)?
  - Head
  - Neck
  - Trunk
  - Front limbs
  - Hind limbs
- F7. How often does your pet experience the myoclonic twitches (specify in twitches per day/month)?
  - Once daily
  - Several times a day
  - Several times a week
  - Once a month
  - Other:
- F8. Does your pet also show generalized tonic-clonic epileptic seizures alongside the myoclonic twitches? (includes rowing movements, unconsciousness, drooling, defecation/urination)
  - Yes
  - No
- F9. What symptoms does your pet exhibit during this time? (e.g., drooling, unconscious loss of urine/feces, loss of consciousness, paddling-stiff limbs, falling to the side, post-seizure disorientation, temporary blindness after seizures)
- F10. Have you received medications from your veterinarian due to the myoclonus seizures?
  - Yes
  - No
- F11. If yes, which medications?
- F12. In which dosage strength and how often per day was the medication administered? If possible, please provide the dosage in mg/kg (milligrams per kilogram of body weight)
- F13. If your dog received medication, did any improvement occur?
  - Yes
  - No
- F14. If improvement occurred, did it manifest as...
  - Reduction in the frequency and severity of myoclonus (twitching)
  - Complete disappearance of myoclonus (twitching)
  - Other:
- F15. How quickly did this improvement occur?
  - After the first dose of the medication
  - After the second dose of the medication
  - After one week with the medication
  - After one month with the medication
  - Other:
- F16. You can send a video of your pet during the episodes to the following email address: kleintierklinik@tiho-hannover.de, with the subject "Dementia and Myoclonus," and consent to the above privacy policy.

# Video Cairn Terrier, positive myoclonus

# Recruitment text for owner questionnaire

Dear dog owners,

Have you ever noticed your beloved furry friend exhibiting unusual muscle twitches/myoclonus or perhaps showing signs of confusion or memory issues? These symptoms could indicate dementia or myoclonus, conditions that can be distressing for both your dog and you. Help us learn more about dementia and myoclonus in dogs! Your experiences and insights are invaluable in improving understanding and treatment of these conditions and potentially gaining life-improving insights.

We warmly invite you to participate in our survey focusing on this topic.

Thank you in advance for your time and support
